# 6-Methoxylated Flavonoids: Jacein, and 3-demethyljacein from *Centaurea schmidii* with Their Endoplasmic Reticulum Stress and Apoptotic Cell Death in Breast Cancer Cells Along with *In-silico* Analysis

**DOI:** 10.22037/ijpr.2020.113895.14548

**Published:** 2021

**Authors:** Mahmoud Aghaei, Mahmoud Mirzaei, Mustafa Ghanadian, Moslem Fallah, Roodabeh Mahboodi

**Affiliations:** a *Department of Clinical Biochemistry, School of Pharmacy, Isfahan University of Medical Sciences, Isfahan, Iran. *; b *Research Institute for Primordial Prevention of Non-Communicable Disease, Isfahan University of Medical Sciences, Isfahan, Iran. *; c *Department of Pharmacognosy, School of Pharmacy, Isfahan University of Medical Sciences, Isfahan, Iran.*; d *Phytochemistry Research Center, Shahid Beheshti University of Medical Sciences, Tehran, Iran. *; e *Department of Chemistry, Faculty of Science, Yasouj University, Yasouj, Iran.*

**Keywords:** Flavonoid, Centaurea, Breast cancer, Endoplasmic reticulum stress, Apoptosis, In-silico analysis

## Abstract

In phytochemical analysis, Jacein derivatives: 5,7,4’-trihydroxy-3,6,3’-trimethoxyflavone-7(β)-D-glucopyranoside (**1**), and 3-demethyljacein: 3,5,7,4’-tetrahydroxy-6,3’-dimethoxyflavone-7(β)-D-glucopyranoside (**2**) were isolated from *Campylopus schmidii (C. schmidii) *for the first time. The structures were determined by interpretation of NMR, UV, and Mass spectra. To check the roles of ER stress and consequent apoptosis in MCF-7 cell by these compounds, UPR signaling pathway was further examined by analysis of expression of ER stress-related genes. In MTT assay, compounds **1**-**2** showed cytotoxicity activity against MCF-7 (A) and MDA-MB cells (B) with IC_50_ values (μM) of **1**) 60.04 ± 7.98 (A), and > 200 (B); **2**) 42.89 ± 1.91 (A), and 85.31 ± 2.68 (B). The Annexin/PI flow cytometry apoptosis of tested compounds **1-2** was increased significantly in a dose-dependent manner. For example, MCF-7 treatment at the concentration of 100 μM of compounds **1**, **2 **resulted in total apoptosis (early + late) of 42.04 (18.1 + 24.0), and 66.49 (2.7 + 63.8)%, respectively. Fluorescence microscopy analysis detected an increased protein aggregation, indicating induced ER stress with a marked increase in XBP-1, sXBP-1, ATF-4, and CHoP compared to untreated cells. *In-silico* characterization, suggested that Adenosine diphosphate site (A-site) and quercetin site (Q-Site) in IRE1a enzyme are both available interacting sites of a target for the investigated ligands but with different strengths of interactions. The results indicated that the ligand∼A-Site complexes are stronger than the ligand∼Q-Site complexes, but the already available ADP ligand in cells does not allow other ligands to interact with the A-Site and cause them to bond in Q-Site.

## Introduction

Recent data of global cancer observation for about 36 cancers from more than 185 countries in 2018 estimates more than 18 million new cases and more than 10 million deaths every year for cancer, from which around 2.05 million (11.39%) suffered from breast cancer. Breast cancer cases are approximately 0.65 million premenopausal and 1·4 million postmenopausal with more than 0.13 and 0.49 million deaths in each group, respectively (1). Reactive oxygen species and inflammatory mediators can cause cellular damages in breast cells which might progressively worsen the situation and increase the risk of malignancies, in particular, if any mutation occurred at the time of cellular repairs. In these situations, ER stress is activated. To restore cellular hemostasis and control the ER stress, adaptive responses named unfolded protein response (UPR) is started. UPR acts through a network of cellular signaling pathways including inositol-requiring enzyme 1 named IRE1, Protein kinase R-like endoplasmic reticulum kinase named PERK, and activating transcription factor 6 (ATF6) (2). If the UPR fails or in the prolonged ER stress criteria, there is a tendency for cellular response to switch from repair or adaptation to programmed cell death to remove damaged cells (3). This process, activation of ER stress, and inducing apoptosis in malignant cells is a new strategy for the treatment of Breast cancer.

In anticancer research studies, *C.* species are highlighted for their cytotoxic activities with low IC_50_ values and high selectivity index values against a panel of cancer cells, including breast cancer cells (4-6). Therefore, *Centaurea schmidii* a native plant that grows wild in Iran, was selected for this study.

Plants belonging to the genus *Centaurea* in the traditional medicine of Iran were used as eyewash in the form of infusion and for rheumatoid pains in the form of oral decoction (7). *C. schmidii* Wagenitz is Basionym of (Wagenitz) Negaresh and is from *Rhaponticoides* sect. *Iranicae*. *C.* *schmidii* is found mainly in temperate regions, sandy slopes, steppes, or spring margins. It is perennial, herbaceous, up to 3 cm in diameter, 70–100 cm long, and with yellowish flowers. It is a rare and endemic species that grows wild in North East of Iran (8). Previously, a guianolide sesquiterpene lactone named: 13‐O‐acetylsolstitialin A has been isolated by the same authors from *C. cyanus* with apoptotic effects against breast cancer cell lines with attenuation of Bax/Bcl‐2 ratio and expression of cyclin D1/Cdk‐4 (9). In the present study, we have isolated and investigated the cytotoxic activity of 6-methoxylated flavonoids like Jacein derivatives isolated from *C. schmidii*. This study revealed the roles of ER stress and consequent apoptosis in MCF-7 cells by these compounds. UPR signaling pathway was further examined by analyzing expression of ER stress-related genes including ATF-4, XBP-1, sXBP-1, TRIB-3, GADD34, and CHOP. 

## Experimental

*General experimental procedures*

High-performance liquid chromatography (HPLC) was done on a Waters HPLC 501 equipped with ultraviolet (UV) and refractive index (RI) detectors and YMC Pack-Sil normal HPLC column (250 × 20 mm, YMC, Japan). The nuclear magnetic resonance (NMR) spectra were captured using an Avance AV400 (Bruker, Germany). Thin layer chromatography was done on Merck silica gel alufoils and visualized by natural flavonoid product (NP) reagent (1% of 2-aminoethyl diphenylborinate in methanol) and ceric sulfate (1 g ceric sulfate, 50 mL H_2_SO_4_, and 500 mL water). Column chromatography was done using Silica gel adsorbents (63-200 μm; 40-63 μm, Merck, Germany) and Polyamide SC6 (Machery Nagel, Germany). Chloroform (ChCl3) and methanol (MeOH) HPLC grade solvents were purchased from Caledon (Canada), and other solvents from Pars chemistry company, Iran, otherwise it is determined in the text. 

Cancer cells were obtained from National Cell Bank of Iran (NCBI). Well plate absorbance (OD) was read using ELISA microplate reader (Bio-Rad, Hercules, CA, USA). The Annexin V-FITC/PI stained cancer cells were counted by a FACS Calibur flow cytometer (BD Bioscience, USA). The amplification of interested gene was performed by Applied Biosystems instrument (ABI 7500 Real-Time PCR System, Foster City, USA). The fluorescence of ThT-protein aggregates was measured at 485/535 nm using Synergy H1 Multimode Microplate Reader. 

*Plant material*

Aerial parts of *C. schmidii* Wagenitz (Asteraceae) was collected from North Khorasan state of Iran at summer at elevation of 1920 m. Plant material was identified by Mohammad Reza Joharchi, Herbarium of Ferdowsi University of Mashhad, Mashhad, Iran where a voucher specimen (FUMH. 45068) was deposited. 

*Extraction and isolation*

**Figure 1:**
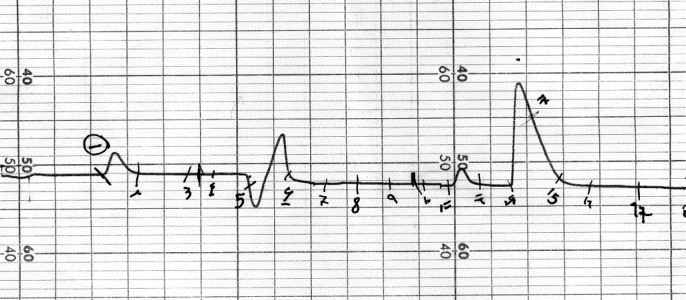
HPLC profile of compound **2** on a YMC silica gel column using ChCl3: MeOH (80:20) with flow rate of 3 mL/min using a refractive index detector

Plant material was dried in shade condition (2200 g), macerated in acetone (10 L) at ordinary temperature for five days, and repeated three times. The resulted extract was concentrated using a rotary evaporator (Heidolph Instruments, Germany) at 40 ºC (230 g). The concentrated extract was partitioned between hexane and aqueous methanol (70:30) in a separating funnel to remove fats and chlorophylls. Methanol partition was concentrated and subjected on silica gel gravity column using a stepwise gradient of hexane: acetone (Fr.1, 90:10; Fr.2, 80:20; Fr.3, 70:30; Fr.4, 50:50; Fr.5, 0:100). Fr.5 with flavonoid pattern in the TLC profile was subjected on polyamide SC6 column using ChCl3: MeOH (Fr.5a, 90:10; Fr.5b, 85:15; Fr.5c 83:17; Fr.5d 80:20). FR.5b, and Fr.5c with the TLC profile of flavonoids were purified by HPLC pump on a silica gel column using ChCl3: MeOH (80:20), with a flow rate of 3 ml/min using RI detector and UV detection at 250 nm (Figure 1). 

*Spectral data*

Compound **1 **(21 mg): ^1^H-NMR (400 MHz, Dioxane D) δ: 3.2-3.8 (H-2^״^- H-6^״^), 3.73 (3H, s, OMe-6), 3.77 (3H, s, OMe-3), 3.84 (3H, s, OMe-3^׳^), 4.97 (1H, d, *J = *7.6, H-1^״^), 6.67 (1H, s, H-8), 6.87 (1H, d, *J = *8.4, H-5^׳^), 7.54 (1H, dd, *J = *2, 8.8, H-6^׳^), 7.63 (1H, d, *J = *2, H-2^׳^). ^ 13^C-NMR (100MHz, Dioxane D) δ: 55.4 (OMe-3^׳^), 59.3 (OMe-3), 59.9 (OMe-6), 61.3(C-6’’), 69.6(C-4’’), 73.5(C-2’’), 77.1(C-3’’), 77.3(C-5’’), 93.7(C-8), 100.7(C-1’’), 107.2(C-10), 111.5(C-5’), 115.2(C-2’), 122.0(C-1’), 122.4(C-6’), 133.1(C-6), 138.4 (C-3), 147.0(C-3’), 149.2(C-4’), 151.7(C-9), 153.3(C-5), 155.8(C-2), 156.2(C-7), 178.8 (C-4). ESIMS molecular ion of [M-H]^-^ at 521 *m/z*.

Compound **2** (15 mg): ^1^H-NMR (400 MHz, Dioxane D) δ: 3.4-3.7 (H-2^״^- H-6^״^), 3.85 (3H, s, OMe-3^׳^), 3.94 (3H, s, OMe-6), 5.11 (1H, d, *J = *7.6, H-1^״^), 6.83 (1H, s, H-8), 6.98 (1H, d, *J = *8.4, H-5^׳^), 7.76 (1H, dd, *J = *2, *J = *8.4, H-6^׳^),7.87 (1H, d, *J = *1.6, H-2^׳^). ^13^C-NMR (100MHz, Dioxane D) δ: 55.3 (OMe-3^׳^), 60.0 (OMe-6), 61.3(C-6’’), 69.6(C-4’’), 73.5(C-2’’), 77.1(C-3’’), 77.3(C-5’’), 93.9(C-8), 100.8(C-1’’), 105.5(C-10), 110.9(C-5’), 115.3(C-2’), 121.8(C-6’), 122.7(C-6’), 132.5(C-6), 136.2 (C-3), 146.4(C-3’), 147.1(C-4’), 145.5(C-9), 151.6(C-5), 152.2(C-2), 156.4(C-7), 146.1 (C-4). ESIMS molecular ion of [M-H]^-^ at 507 *m/z*.

*Cell culture*

Two different human breast cancer cell lines, MCF-7 as estrogen receptor positive, and MDA-MB 231 as estrogen receptor negative, were obtained from National Cell Bank of Iran (NCBI). The cells were cultured in RPMI 1640 medium with 10% fetal bovine serum (FBS), contained penicillin (100 u/mL), and streptomycin (100 μg/mL), in a 5% CO2 incubator at 37 °C (9).

*Cell viability assay*

Cell viability was determined by MTT assay as described previously (9). Briefly, the cells with a density of 5 ×10^3^ cells /well were seeded into 96-well plates in RPMI media and incubated overnight (5% CO2, 37 °C). Then new media was replaced, and tested compounds **1-2** in the concentrations of 1, 10, 25, 50, 100, 200 μM, Taxol (Ebewe Pharma, Austria) in the concentrations of 0.001, 0.01, 0.1, 1, and 10 μM, and corresponded solvent for each concentration as negative control were added and incubated for next 48 h. Then 20 μL of MTT (5 mg/ml in PBS) was added to wells incubated again for 4 hr. at the same conditions. Then supernatants were eliminated, biological grade DMSO was added, and the absorbance (OD) was read at 570 nm in an ELISA microplate reader (9).

*Annexin V-FITC/PI assay of apoptosis*

Apoptotic cell death induced by compounds was quantified by flow cytometry using the Annexin V-FITC/PI kit (10). Briefly, MCF-7 cells were seeded in a six-well plate by the density of 3×10^5^ per well and treated with tested compounds (0.1, 1, 10, and 100 µM) for 24 h in an incubator (5% CO2, 37 °C). Floated and attached cells were washed twice with PBS and suspended again in binding buffer. Then using Annexin V-FITC/PI kit protocol, Annexin V-fluorescein isothiocyanate/ PI (each 5 µL) were added to wells to stain the cells and incubated again for 10 min at room temperature. The stained cancer cells were counted by a FACS Calibur flow cytometer, and the results analyzed using the software in the instrument (10).

*Endoplasmic reticulum stress assay by Thioflavin staining and fluorescence microscopy analysis*

ER stress detection by Thioﬂavin T (ThT; Sigma Aldrich, USA) staining of unfolded and aggregated proteins was used to check MCF-7 cellular ER stress. MCF-7 cells were seeded in a density of 3 × 10^5^ per well in a 24-well plate and incubated by adding tested compounds in the concentrations of 1, 10, and 100 µM, and Tunicamycin (2 μg/mL) as positive control for 24 h. Untreated cells were regards as negative control. ThT (5mM) was added to wells, and after half of an hour incubation (37 °C), Triton in PBS (0.1%) was added, and using a cell scraper, cells were lysed. Cell lysates (100 mL) in each well were transferred into a black-bottomed well plate special for the ELISA ﬂuorescence reader, and examined ThT stained protein aggregates at 485 nm excitation and 535 nm emission spectral condition. ImageJ software was used to analyze, and measure the relative fluorescent signals (10).

*ER Stress Gene Expression*

ER stress gene expression was done as described before (10). Briefly, MCF-7 Cells were exposed for 24 h to tested compounds **1-2** in the concentrations of 1, 10, and 100 μM, Tunicamycin (2 μg/mL) as positive, and using GAPDH (Glyceraldehyde 3- Phosphate Dehydrogenase) as an internal control. Total RNA was extracted by Trizol (Sigma-Aldrich, USA), and its purity and concentration were checked by UV spectrometer. RNA reverse transcription was done using Qiagen kit (Qiagen, UK), and cDNA was extracted and subjected to real-time quantitative PCR (50 °C for 2 min, 95 °C for 10 min, 40 cycles at 95 °C for 15 s and 60 °C for 1 min). The sequences of XBP-1, sXBP-1, ATF-4, TRIB-3, GADD34, and CHOP primers are mentioned in Table 1. Using Excel software, all the real-time gene expression levels were normalized and validated by GAPDH. Finally, the mRNA expressions were reported using the 2^-ΔΔCt^ analysis (3).

*In-silico* *analysis*

IRE1α is a protein with RNase function which cut X-box-binding protein 1 (XBP1) mRNA in response to ER stress and made its active spliced form (sXBP1), leading to UPR (2). Fohlen *et al*. showed that XBP1 splicing is done only by IRE1α. Therefore, the marked increase in sXBP1 in this study encouraged us to check the binding affinity of these compounds on active sites of IRE1. IRE1 has one active site named A-site and another binding site for substrate mRNA (11) required for RNase activity. Adenosine diphosphate (ADP) can bind to A-site as an indigenous endoribonuclease activator of IRE1 (12). Luke Wiseman et ql. reported that quercetin (QUE) with flavonol structure bind on another binding site named Q-site in the presence of ADP in A-site. They made a co-crystal of IRE1 with ADP in A-site and quercetin in Q-site and reported that quercetin enhances ADP activity while stabilizing also a dimeric form of IRE1 (13). Compound **1-2** also has a flavonoid structure, and considering it, we performed computational docking and *in-silico *analysis to check if tested flavonoids can act like QUE on IRE1 and enhance RNase activity. In the first, the model structures have been optimized to the minimum energy by the Gaussian 09 program (14) under the B3LYP/3-21G* standard theoretical level of density functional theory (DFT). To approve non-imaginary frequency existence, frequency calculations have been done at the same theoretical level, and the structures have been found valid. As a result, three ligands have been prepared for further investigations by the Molecular Docking simulations (MDs). In the next step, the 3LJ0 PDB structure of IRE1 complexed with adenosine diphosphate (ADP) and Quercetin (QUE) (13) was obtained from Protein Data Bank (id: 3LJ0), and it was prepared as a target for MDs by eliminating ADP, and QUE. Furthermore, the Kollman charge has been added to the hydrogenated PDB. The grid box has been set to 40 × 40 × 40 once for docking of A-Site and once again for docking of Q-Site with assigned 100 numbers of genetic algorithm (GA) conformational search as implemented in the AutoDock4 program for each docking process. In addition, Gasteiger charges were added to each of the ligand structures to be prepared for MDs processes (14).

## Results

*Identification of flavonoids*

Compound **1**, was obtained as a pale solid with a positive reaction to natural product reagent and ESIMS molecular ion of [M-H]^-^ at 521 *m/z*. The UV spectrum showed absorption maxima at 258.6, 352, and a shoulder at 272 nm, without bathochromic shifts of Band I in AlCl3/HCl at pH of 3 but with 41 nm bathochromic shifts of Band I in AlCl3 at 398 nm in pH of 7 indicative of the presence of a chelating hydroxyl group at C-5 in flavonol structure (15). The ^1^H-NMR showed resonances of an *ortho *coupled proton of H-5’, and H-6’ signals at δ_H_ 7.54 (1H, dd, *J* = 8. 4, 2.0 Hz) and 6.87 (1H, d, *J* = 8.4 Hz), and *meta* proton coupling of H-2’ at δ_H_ 7. 63 (1H, d, *J* = 2.0 Hz) indicative of ABX system in ring B, in addition to one singlet proton at δ_H_ 6.67 (s) related to H-8 which was indicative of a 3,5,6,7,3’,4’-hexahydroxyflavonol derivative (15). BB and DEPT spectra indicated the presence of three methoxy moieties: *δ*_C _59.9 (*δ*_H_ 3.73 s, 6-OMe), 59.3 (*δ*_H_ 3.77 s, 3-OMe), and 55.4 (*δ*_H_ 3.84 s, 3’-OMe), one glycosyl part:* δ*_C _100.7 (GLC-1’’), 77.3 (GLC-5’’), 77.1 (GLC-3’’), 73.5 (GLC-2’’), 69.6 (GLC-4’’), and *δ*_C _61.3 (GLC-6’’), and a flavonol core comprised of 4 sp2 methin, 11 quaternary carbons attributable to one carbonyl, and 10 aromatic quaternary carbons from which 8 were oxygenated (see spectral data). HMBC placed methoxy groups and determined glycosylic linkage at C-7 through HMBC correlations between δ_H_ 4.97 (d, *J* = 7.6, H-1’’) and quaternary olefin carbon C-7 (δ_C_ 156.2). The configurations of anomeric proton was deduced to be *β* based on coupling constants (*J* = 7.6 Hz). Therefore, compound **1** was deduced to have a structure of 5,7,4’-trihydroxy-3,6,3’-trimethoxyflavone-7-O-β-D-glucopyranoside called Jacein (16). 

**Figure 2:**
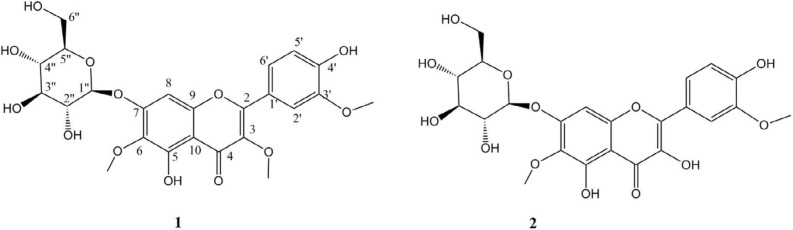
6-Methoxylated flavonoids from *C. schmidii*

Compound** 2** was yielded as a yellowish solid with ESIMS molecular ion of [M-H]^-^ at 507 *m/z*. The UV spectrum showed absorption maxima at 257 and 373 nm, with bathochromic shifts of Band I in AlCl3 with 54 nm shifts of Band I in AlCl3 at 427 nm indicative of the presence of free 3- and 5-OH in flavonol structure (16). The ^1^H- and ^13^C-NMR showed resonances similar to those reported in **1** except for the loss of 3-O- methoxy at *δ*_C _55.4 (*δ*_H_ 3.77 s). Therefore, it was determined as 3-demethyljacein or 3,5,7,4’-tetrahydroxy-6,3’ -dimethoxyflavone-7-O-β-D-glucopyranoside (Figure 2). Its structure and spectral data were previously reported by Olennikov et al from *Gnaphalium uliginosum* at 2015 (17).

*MTT viability assay against MCF-7 and MDA-MB Human breast cancer cells*

**Figure 3:**
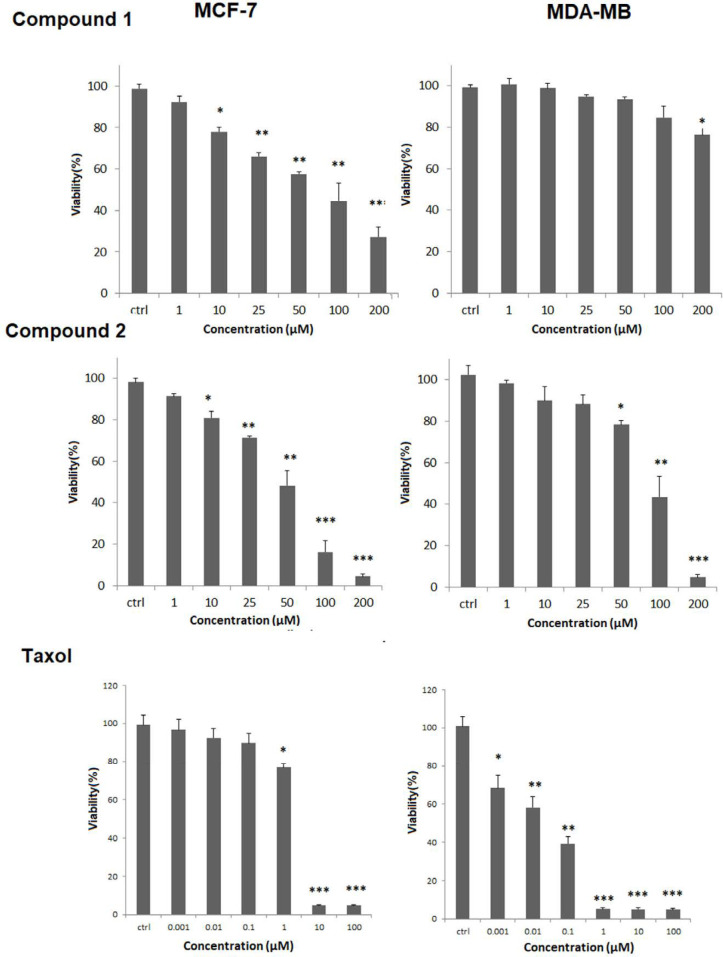
Compounds **1-2** cytotoxicity effect using MTT viability assay against a) MCF-7, and b) MDA-MB231cells. Tested compounds were used in 1, 10, 25, 50, 100 and 200 µM and in three replicates. Results as mean ± standard deviation (SD) were reported as percent of control values. (^*^p < 0.5; ^**^p < 0.01; ^***^p < 0.01 *versus* control)

The effect of compound**s 1**-**2** on cell viability was carried out by MTT assay**.** As shown in Figure 3, MCF-7 and MDA-MB-231 cell lines were incubated with different concentrations of compounds **1-2** for 24 h. They showed promising activity in a dose-dependent reduction in the cell viability with IC_50_ values of 60.04 ± 7.98, and 42.89 ± 1.91 μM against MCF-7 cells but weak cytotoxicity against MDA-MB231 cells with IC_50_ values more than 200 μM for compound 1, and 85.31 ± 2.68 μM for compound **2**. The IC_50_ of Taxol as standard drug was 4.37 ± 0.12 and 0.048 ± 0.0098 μM against MCF-7 cells, and MDA-MB231, respectively.

*Annexin/PI flow cytometry assay of apoptosis*

**Figure 4:**
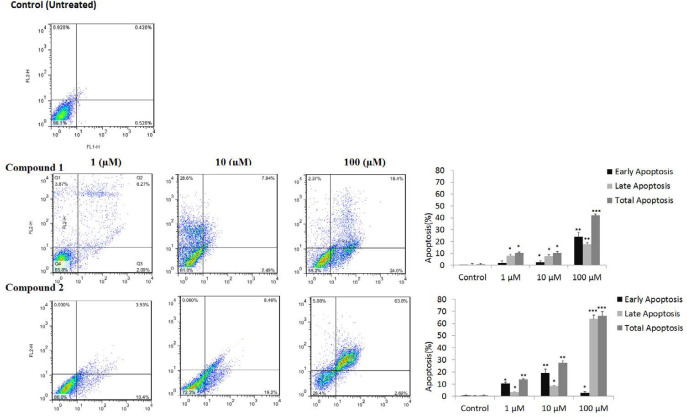
Compounds **1-2** showed apoptosis in MCF-7 carcinoma cells. Cells were treated with 1, 10 and 100 μM concentrations for 24 h, stained with Propidium iodide (PI) and annexin-V and examined by flow cytometer. (^*^p < 0.5; ^**^p < 0.01; ^***^p < 0.01 *versus* control)

Estrogen-dependent MCF-7 cells were more sensitive to compounds **1-2 **than non-estrogenic MDA-MB 231 breast cancer cells. Therefore, MCF-7 cells were selected for Annexin/PI flow cytometry assay of apoptosis to determine whether growth-inhibitory effects are through programmed cell death. MCf-7 cells were analyzed by flow cytometry using FITC-conjugated annexin V (FL1-H) and PI (FL2-H) double staining to differentiate between lived normal cells (Q3: Annexin V−/PI−), early apoptosis (Q4: Annexin V+/PI−), late apoptosis (Q2: Annexin V+/PI+), and necrotic cells (Q1: Annexin V−/PI+). As clear from Figure4, total apoptosis (Q3+Q2) of tested compounds **1-2**, is increased significantly in a dose-dependent manner. For example, MCF-7 treatment at the concentration of 100 μM of compounds **1**, **2 **resulted in total apoptosis of 42.04 (18.1 + 24.0), and 66.49 (2.7 + 63.8)%, respectively. 

*Fluorescence Microscopy Analysis and Endoplasmic reticulum stress assay*

**Figure 5:**
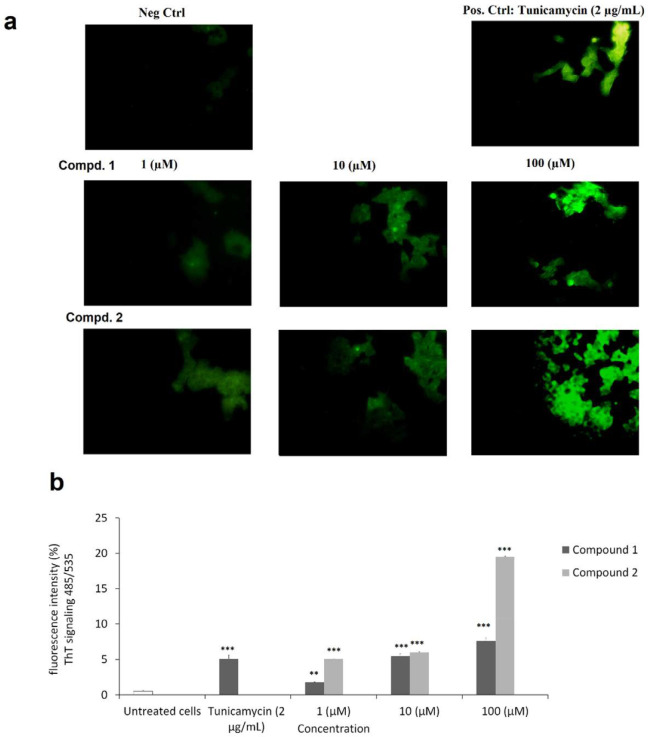
Endoplasmic reticulum stress assay detection by Thioﬂavin T (ThT) staining of protein aggregation and microscopic fluorescence analysis. a) Live MCF-7 cell images of ThT-protein aggregates were taken using 485 nm excitation and 535 nm emission. b) The fluorescence of ThT-protein aggregates was analyzed by Image J application and presented as mean ± SD. (^*^p < 0.5; ^**^p < 0.01; ^***^p < 0.01 *versus* control)

As clear in Figure 5a, by fluorescence microscopy analysis using ThT staining, we first detected changes in protein aggregation and found a rapid increase in ER stress within 24 h, indicating induced ER stress in MCF-7 cells (Figure 5a). Then using Image J application, fluorescent intensity at concentration (1, 10, and 100 µM) of the compounds **1-2** were quantified compared to negative control and Tunicamycin as standard drug (Figure 5b). Results showed a dose dependent manner increase in fluorescent intensity of ThT stained aggregated proteins (485/535 nm) with significant increase from lower concentration 1 µM (*p* < 0.05). Tested compounds **1-2** at higher concentration of 100 µM showed fluorescent intensity of ThT signaling by 7.57 ± 0.43, and 19.53 ± 0.93%, respectively, while Tunicamycin (2 µg/mL) showed 5.07 ± 0.55%, as positive control.

*Endoplasmic reticulum Stress corresponded Gene Expression*

**Figure 6:**
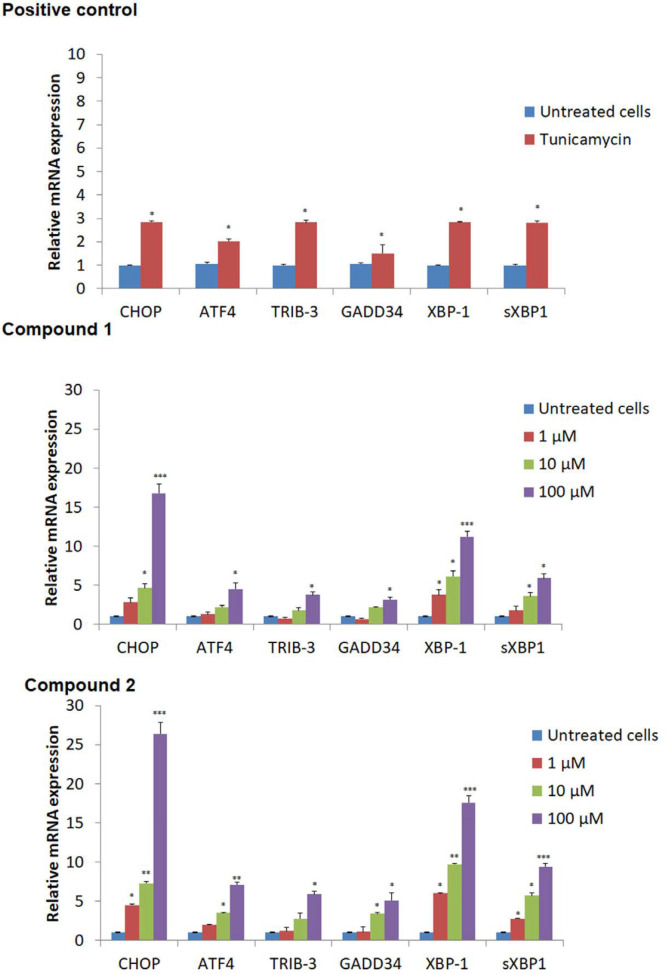
mRNA expression of important signaling proteins in endoplasmic reticulum UPR signaling. The expression of each mRNA relative to GAPDH was calculated by ΔΔC (t) method. Vehicle was used as negative and Tunicamycin as positive controls. (^*^p < 0.05; ^**^p < 0.01; ^***^p < 0.001 *versus* negative control)

We next evaluate the effects of compounds 1-2 on ER stress-related gene expressions such as ATF-4, XBP-1, sXBP-1, TRIB-3, GADD34, and CHoP, which were studied using RT-PCR analysis. As displayed in Figure 6, after 24 h treatment of compounds, mRNA expression of ATF-4, XBP-1, sXBP-1, TRIB-3, GADD34, and CHoP genes increased compared to untreated cells. These findings and fluorescence microscopy analysis and endoplasmic reticulum stress assay, confirmed that compounds **1-2** induced ER stress in breast cell lines. Compound 2 showed more activity with 26.4 ± 1.5, 7.1 ± 0.35, 5.9 ± 0.35, 5.1 ± 1.01, 17.6 ± 82, and 9.4 ± 0.45 fold of control relative mRNA expression for CHoP, ATF4, TRIB-3, GADD34, XBP-1, and sXBP-1 proteins, respectively at higher concentration of 100 μM (Figure 6).

*In-silico characterization*

**Figure 7:**
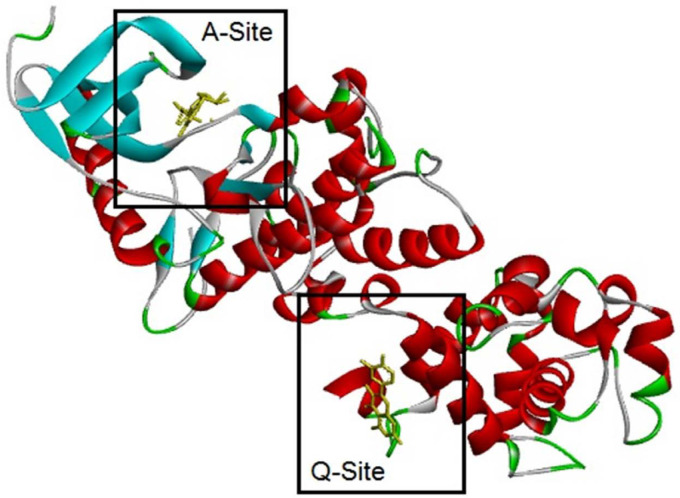
IRE1 target enzyme complexed adenosine diphosphate (ADP) and Quercetin (QUE). 3LJ0 PDB structure of with IRE1 complexed adenosine diphosphate (ADP) and Quercetin (QUE) was obtained from Protein Data Bank (id: 3LJ0) and it was prepared as target for MDs by eliminating ADP, and QUE

It should be mentioned that two sites of the target are essential, A-Site and Q-Site, in which the A-Site is specified by the position of ADP and the Q-Site is specified by the position of QUE in the downloaded crystal structure of the IRE1 enzyme (Figure 7). Therefore, ligands were eliminated, and docking processes were done on active sites. 

In one of the experiments, compounds **1-2** and ADP were docked in the A-Site (ADP site). In parallel experiment compounds, **1-2** and QUE were docked in Q-Site (Quercetin site). In the docking process of the Q-Site, ADP was already fixed in its original position of A-Site. Quantitative values of binding energies (EB) and inhibition constants (KI) were evaluated for the docked complexes in addition to qualitative representations of ligand-target interacting complexes. 

In the first experiment, all ligands were individually docked in the pre-defined A-Site of the target enzyme, in which the scoring factor based on EB shows that ADP is the best for this position. However, in the absence or low concentration of ADP, compounds **1-2** have a chance to be docked in the A-Site even better than QUE, which previously is reported to have an affinity with this site (13). The trend means that although ADP is the best one for the A-Site, in the lack of ADP, A-site could provide an available interacting position for ligands **1-2**. 

**Figure 8:**
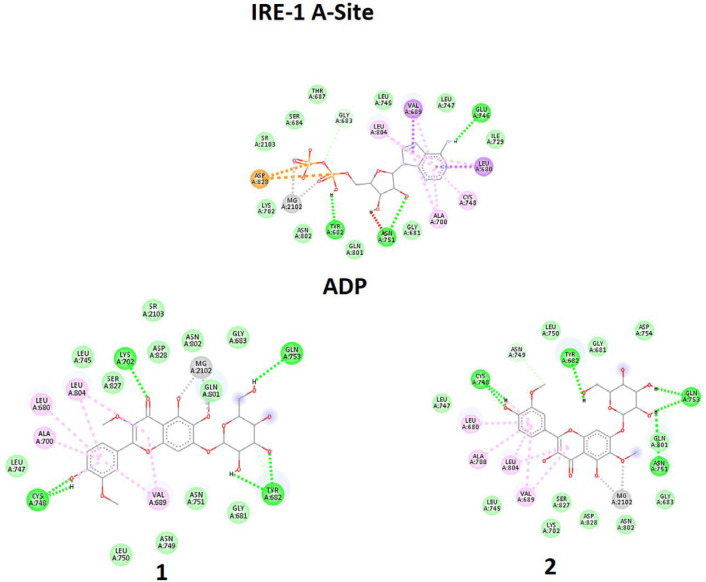
Molecular docking simulation of ligands **1-2**, and Adenosine diphosphate (ADP) with A-site of the IRE1 target enzyme with Protein Data Bank ID of 3LJ0. Ligand structures were optimized to the minimum energy by the Gaussian 09 program under the B3LYP/3-21G* standard theoretical level of density functional theory. The grid box has been set to 40 × 40 × 40 once for docking of A-Site with assigned 100 numbers of genetic algorithm conformational search for each docking process

Qualitative analysis of interacting amino acids (Table 2 and Figure 8) could also approve the trend, in which very good similarities are seen for the interacting counterparts of compounds **1-2** with those of ADP. 

**Figure 9:**
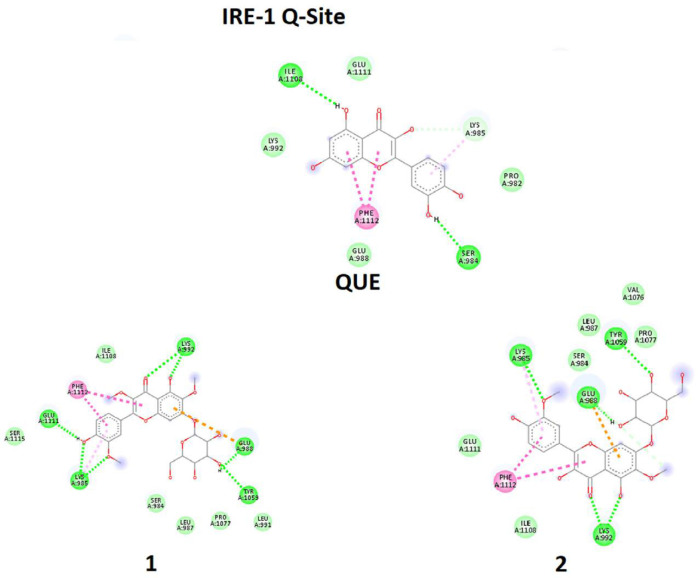
Molecular docking simulation of ligands **1-2**, and Quercetin (QUE) with Q-site of the IRE1 target enzyme with Protein Data Bank ID of 3LJ0. Ligand structures were optimized to the minimum energy by the Gaussian 09 program under the B3LYP/3-21G* standard theoretical level of density functional theory. The grid box has been set to 40 × 40 × 40 once for docking of A-Site with assigned 100 numbers of genetic algorithm conformational search for each docking process

In the second experiment in binding to Q-Site, ligands **1-2** and QUE were docked in the Q-Site of target enzyme to see the potency of ligands for interaction with the site in the presence of ADP but fixed in A-Site, which is essential for dimerization of IRE1 and RNase activation. The results (Table 2, Figure 9) indicated that compounds **1** - **2** could be implemented in Q-site even with more affinity than QUE for interacting with this site. The values of BE and KI could approve the trend. Quercetin binding to this site potentiates IRE1 activity. Therefore these compounds with similarities to quercetin structure are supposed to have the same effect.

In comparison between A-site, and Q-site, the strength of interaction for flavonoid ligands of Q-Site is weaker than those of A-Site, meaning that the ligands have more affinity for the A-Site than the Q-Site, but the pre-fixed ADP does not allow the ligands to be docked in the A-Site. While in Q-site, qualitative analysis of interacting amino acids also showed that both compounds **1-2** had interactions similar to QUE regarding the interaction environment but with different strengths because of interaction types.

Luke Wiseman *et al*. reported that quercetin with flavonol structure binds on the Q-site of IRE1. They made a co-crystal of IRE1 with ADP in A-site and quercetin in Q-site and reported that quercetin helps make a dimer form of IRE1 with more RNase activity than IRE1 activated by ADP alone (13).Compounds 1, and 2 possessed also the flavonoid structure and might have the same mechanism. Therefore, we performed computational docking and *in-silico *analysis to check if tested compounds can act like QUE on IRE1 and enhance RNase activity in dimer form. Dimerization analyses for ligand compounds have been performed based on DFT calculations to evaluate the most possible paring configuration of ligands (19-20). The configuration representation shows that after dimerization, the molecular sites would be almost involved in homo paring interactions (Only one free hydroxyl group on C-4’), lowering the possibility of compound **1** to interact with other molecules leading to its less activity in ER stress, while in the case of compound **2**, more atomic and molecular sites (free hydroxyl groups on C-3 and C-4’) are still free and contribute in further interactions which enhance its activity. The configuration representation also shows that the paired system is in a perpendicular mode, and almost all atomic and molecular sites are free for contributing to further interactions. For QUE almost similar situations are seen. As a remarkable achievement of these analyses, it could be mentioned that the dimer of compound **2** is the best candidate for interaction with other molecular systems among the investigated model systems. 

## Discussion

Tested compounds, especially compound **2** in a dose-dependent manner and exponential scales reduced viabilities against breast cancer cells. They showed more selectivity against estrogen receptor-positive MCF-7 than estrogen negative MDA-MB23 cells. These results agree with previous studies on anticancer activity of other flavonoid structures against breast cancer and their anti-aromatase and anti-estrogenic activities in ER positive MCF-7 cells (21- 23). MCF-7 cells were selected for more analysis by flow cytometry, ER stress assay, and gene expression. Flow cytometry using the annexin V-FITC kit confirmed apoptotic cell death in MCF-7 cells induced by compounds **1-2**. As clear from Fig 4, cytotoxicity was mostly through apoptosis, and necrotic cell death was ignorable even at a higher concentration of 100 μM. Compound **2** showed more activity than **1,** with more apoptosis (66.49% including 2.7% early and 63.8% late apoptosis), and less necrotic cell death (5.08%) at 100 μM.

ER stress is one of the potential molecular mechanisms of apoptosis. ER stress responses are changes in cellular homeostasis that inhibit protein expression, folding and even accumulating malfolded proteins in the ER (24). ER stress assay indicated induced ER stress by tested compound **1-2** in MCF-7 cells started from lower concentration. Protein aggregation induced by ER stress was detected by Thioﬂavin T (ThT) staining, fluorescence microscopy analysis, and consequent analysis using ImageJ application. It showed more activity for compound **2** at 100 μM treatment with 19.53 ± 0.93% fluorescence intensity of ThT signaling (485/535 nm) than compound **1**in the same condition. Under ER stress situations, unfolded protein response named UPR controls the folding of proteins in the ER. UPR consequences in cells are caused at first adaptation, and if adaptation fails or under prolonged stress, it induces apoptotic cell death (2). ER stress activates three UPR branches with a wide variety of signaling pathways include PERK, IRE1, and ATF6, which could be determined through the detection of involved proteins or their mRNA expressions (3). In this study activity of tested compounds made dose-dependent increase in gene expression of ATF-4, XBP-1, sXBP-1, TRIB-3, GADD34, and CHOP, which are involved in UPR and were evaluated using real-time RT-PCR. As clear in Figure 6, compounds **1-2** increased XBP1, sXBP1, and CHOP of *IRE1 *pathway in UPR. IRE1α is a protein with endoribonuclease function which cut an intron from X-box-binding protein 1 (XBP1) mRNA, and made its active spliced form (sXBP1) which binds to promoters, and activates downstream genes involved in UPR (2). Fohlen B *et al.* showed that XBP1 splicing is done only by IRE1α ribonuclease activity, and when ER stress occurs, marked increase in sXBP1 confirms ER stress activation in IRE1 branch (3, 25). IRE1 activates particularly mitogen-activated protein kinase 5 (MAP3K5), which is ASK1 or apoptosis signal-regulating kinase 1, and consequently leads to activation of c-Jun N-terminal kinases (JNKs) (26). IRE1 may also activate the BAX or BCl-2-associated X proteins to induce apoptosis. Another UPR signaling protein is transcription factor C/EBP homologous protein (CHoP) as a downstream protein in all three ER-stress branches, which contains binding sites for ATF4, ATF6, and XBP1 (26). As clear from Fig 6, ATF-4 is increased in a dose-dependent manner (*p* < 0.05). ATF4, and CHOP have an upregulation effect on the transcription of growth arrest and DNA-damage-inducible protein 34 (GADD34), which was confirmed by its increase in a dose-dependent manner. 

This study agrees with recent emerging studies on other types of flavones like kaempferol, and apigenin on ER stress. Previously, quercetin with flavonol structure was reported to bind to the Q-site in IRE1 protein and activates its endoribonuclease activity leading to cleavage of XBP1 to spliced XBP1 mRNA and activate UPR signaling (13). Kaempferol, another flavonol structure, was reported to have modulatory effects on UPR signaling. It induces cell death by activating UPR and CHOP protein in cancer cells, while the same compound restores cell survival through CHOP suppression and GRP78 in normal noncancerous cells (27). Abdullah *et al*., reported that kaempferol is docked in the same position in which adenosine diphosphate interacts in the kinase domain of IRE1α enzyme, and could activate the RNase activity of IRE1 consequently (12). 

Wiseman *et al.* reported that the arrangement of two quercetin molecules in the Q-site of each strain of IRE1 dimer helps bridge the two protomers through pi-pi or hydrogen binds (13). Compounds **1-2** also possess flavonoid structures like Kaempferol and quercetin and act by similar function, which was confirmed by autodock results. ER stress, and apoptosis results showed that compound **2** has more activity than compound **1**, probably due to the presence of free hydroxyl group at C-3 position, which agrees with Wiseman et al report on quercetin and other flavonoids. The *in-vitro* comparison of IRE1 RNase activity of 3,5,7,3′,4′-pentahydroxyflavonol (quercetin) with other flavonoids including 3,5,7,4′,-pentahydroxyflavonol (kaempferol), 3′-methoxy-3,5,7,3′,4′-pentahydroxy flavonol (isorhamnetin), 3,5,7,2′,4′-pentahydroxy flavonol (Morin), 5,7,3′,4′ tetrahydroxyl flavone (Luteolin), and 5,7,4′-trihydroxyl flavone (apigenin) in the presence of ADP showed activity in the following order: Quercetin = Kaempferol > Luteolin > Isorhamnetin > Morin>ADP supports quercetin or kaempferol more activities because of possessing C-3 free hydroxyl group, even though oxygenation pattern in monocyclic ring B specially C-4′ hydroxyl group should be also considered (13).

Our study can be compared with other natural products with ER stress-related apoptotic cell death. For example, Garrido-Armas *et al.* reported that curcumin induces cell death by apoptosis pathway involving the ER in A172 human glioblastoma cell line. In this study, alteration is occurred in mRNA expression of ATF6 and IRE1α, miR-449, miR-222, and miR27a proteins (28). Chang LC *et al.* demonstrated that a curcumin derivative induced apoptosis ER stress in the MDA-MB-231 breast cancer cells by changes in ER stress genes PERK, PDI, CHOP, calnexin, ERO1, and BiP ER (29). Chow *et al.* showed that resveratrol, a phenolic compound with a stilbene structure found in red grapefruits, has apoptotic effects against nasopharyngeal carcinoma cell lines (NPC-TW076 and NPC-TW039). It induces ER stress along with autophagy by the marked increase of p-PERK, IRE1, CHOP, and ATF6 mRNA expressions (30). Davalli P *et al.* reported that Green tea extract rich in flavanols and epigallocatechin gallate has prominent cytotoxic activity against PC3 prostate cancer cells and induces ER stress through PERK branch signaling by an increase in p-eIF2α and ATF4 genes (31). Krajarng A et al showed that Gambogic acid, a prenylated xanthonoid compound from *Garcinia hanburyi*, causes ER stress in HeLa cancer cells by altering ER stress genes (32). Aghaei reported that a sesquiterpene from *Pimpinella haussknechtii* caused apoptotic death through activation of PERK branch by mark increase of ATF4, followed by an increase in CHoP and GADD34 in MCF-7 breast cancer cells (10). 

These results agree with another study done by Han et al showing that Hispidulin has a pro-apoptotic effect against human hepatocellular carcinoma cells (SMMC7721) via ERS mainly by increasing CHOP protein. They also reported more details on the signaling pathway of hispidulin apoptosis induction through AMPK/mTOR pathway. It also showed in their study that 25 and 50 mg/kg of Hispidulin in HCC xenograft nude mice (ip) for one month has anti-tumor activity with increasing ERS protein expression (GRP78, CHOP) along with a decrease in expression of Bcl-2 in tumor tissues (33).

Lv *et al.* also reported that Hispidulin reduced NCIH460 human non-small cell lung cancer cell growth in mouse xenograft model through the generation of ROS. They showed that Tauroursodeoxycholic acid, a bile acid with specific ER stress inhibitory effect reversed antitumor effects and suggested that Hispidulin exerts its apoptotic effect via ROS generation and ER stress activation pathways (34).

The above mentioned results of previous studies and our results confirm the endoplasmic reticulum stress and apoptotic cell death properties of Jacein and 3-demethyljacein in MCF-7 Breast cancer cells. However, confirmation of efficacy through other *in-vitro* studies, including anti-aromatase activities, and different cytotoxic tests against breast cancer, in addition to *in-vivo* hormone-positive breast anti-tumor efficacy in athymic nude mice, are required to get more details on the anti-tumor activity of these two compounds against ER positive breast cancer cells.

**Table 1:** Primer sequences of ERS gene markers which were used for RT-PCR.

Genes	Accession number	Forward primer	Reverse Primer
GRP78, BIP, HSPA5	NM_001163434	ATGGACCTGTTCCGCTCTAC	GCTCCTTGCCATTGAAGAAC
CHOP, Ddit3	NM_007837	CCAGGAAACGAAGAGGAAGA	TCTGACTGGAATCTGGAGAGC
XBP1	NM_013842	AACACGCTTGGGAATGGAC	GTGCACATAGTCTGAGTGCTG
sXBP1	NM_001271730	CTGAGTCCGCAGCAGGTG	AGCAGACTCTGGGGAAGGAC
ATF4	NM_009716 NM_001287180	ATGGGTTCTCCAGCGACA	GAAAAGGCATCCTCCTTGC
GAPDH	NM_008084 NM_001289726	GTCGGTGTGAACGGATTTG	AGGTCAATGAAGGGGTCGT

**Table 2:** Docking properties^*^.

	**1**	**2**	**QUE**	**ADP**
**A-Site**				
EB	-8.46	-7.93	-7.31	-11.87
KI	63	153	438	19
AA	LEU680 GLY681 TYR682 GLY683 VAL689 ALA700 LYS702 LEU745 LEU747 CYS748 ASN749 LEU750 ASN751 GLN753 GLN801 LEU804 SER827 ASP828 MG2102 SR2103	LEU680 GLY681 TYR682 GLY683 VAL689 ALA700 LYS702 LEU745 LEU747 CYS748 ASN749 LEU750 ASN751 GLN753 ASP754 GLN801 ASN802 LEU804 SER827 ASP828 MG2102	GLY683 SER684 VAL689 LEU680 GLY681 TYR682 ALA700 LEU747 CYS748 ASN749 LEU750 ASN751 ASP754 GLN801 ANS802 LEU804 ASP828 MG2102 SR2103	LEU680 GLY681 TYR682 GLY683 THR687 VAL689 ALA700 LYS702 ILE729 LEU745 GLU746 LEU747 CYS748 ASN751 GLN801 ASN802 LEU804 ASP828 MG2102 SR2103
**Q-Site**				
EB	-4.17	-3.31	-4.11	-4.02
KI	874	3840	991	1130
AA	SER984 LYS985 GLU988 LEU991 LYS992 LEU987 TYR1059 PRO1077 ILE1108 GLU1111 PHE1112 SER1115	SER984 LYS985 LEU987 GLU988 LYS992 TYR1059 VAL1076 PRO1077 ILE1108 GLU1111 PHE1112	PRO982 SER984 LYS985 GLU988 LYS992 GLN1107 ILE1108 GLU1111 PHE1112	PRO982 SER984 LYS985 GLU988 LYS992 ILE1108 GLU1111 PHE1112

^*^Binding Energy (EB) is in kcal/mol; Inhibition constant (KI) is in µM; AA are interacting amino acids with each ligand.

## Conclusion

In MCF-7 human breast cancer cell lines, 3-6-methoxylated flavonoids: Jacein and 3-demethyljacein from *C. schmidii* induced ER stress related apoptotic cell death by increasing protein aggregation as demonstrated by fluorescence microscopy analysis and by the marked increase in gene expression of XBP1, sXBP1, ATF-4, and ChoP proteins. *In-silico *characterization suggested that A-Site and Q-Site are both available interacting sites of target for the investigated ligands but with different strengths of interactions. The results indicated that the ligand∼A-Site complexes are more substantial than the ligand∼Q-Site complexes, but the already available ADP ligand in cells does not allow other ligands to interact with the A-Site and underlie them to Q-Site. 
